# Rheumatoid arthritis: a comprehensive overview of genetic markers, emerging therapies, and personalized medicine

**DOI:** 10.1097/MS9.0000000000002890

**Published:** 2025-01-07

**Authors:** Laiba Shakeel, Ayesha Shaukat, Nawal Khaliq, Aayat Kashif, Azka Mujeeb, Zahabia Adnan, Javeria Taj, Aymar Akilimali

**Affiliations:** a Department of Internal Medicine, Dow University of Health Sciences, Karachi, Pakistan; b Department of Internal Medicine, Jinnah Sindh Medical University, Karachi, Pakistan; c Department of research, Medical Research circle, Goma, Democratic Republic of the Congo

**Keywords:** artificial intelligence, autoimmune disease, biologic therapies, dMARDs, genetic markers, inflammatory arthritis, personalized medicine, rheumatoid arthritis, telemedicine

## Abstract

Rheumatoid arthritis (RA) is a prevalent autoimmune disorder marked by chronic inflammatory arthritis and systemic effects. The etiology of RA is complex, involving genetic factors like HLA-DR4 and HLA-DR1, as well as environmental influences, particularly smoking, which heightens disease risk. Affecting approximately 1% of the global population, RA is associated with considerable morbidity and mortality, with its prevalence expected to increase due to demographic shifts, especially in certain regions. RA symptoms commonly manifest between ages 35 and 60 but can also affect children under 16 in cases of juvenile RA. Symptoms include prolonged joint stiffness, pain, fatigue, and, in advanced cases, joint deformities. Current treatment approaches involve disease-modifying antirheumatic drugs, biologics, and glucocorticoids to manage symptoms and slow disease progression, though these treatments often present limitations due to adverse effects and varied patient response. The identification of genetic markers, such as HLA-DRB1 and PTPN22, supports the growing emphasis on personalized treatment strategies that account for genetic and lifestyle factors. Non-pharmacological approaches – diet adjustments, physical activity, and stress management – are increasingly valued for their complementary role in RA management. Lifestyle interventions, including whole-food, plant-based diets and physical therapy, show promise in reducing inflammation and improving joint function. Technological advancements, like telemedicine, mobile health applications, and artificial intelligence, are enhancing RA diagnosis and treatment, making care more precise and accessible. Despite these advancements, RA remains incurable, necessitating continued research into novel therapeutic targets and approaches. A comprehensive, patient-centered approach that integrates lifestyle modifications, preventive strategies, and innovative treatments is essential for improving RA management and patient outcomes.

## Introduction

### Background of rheumatoid arthritis

**Figure 1.**
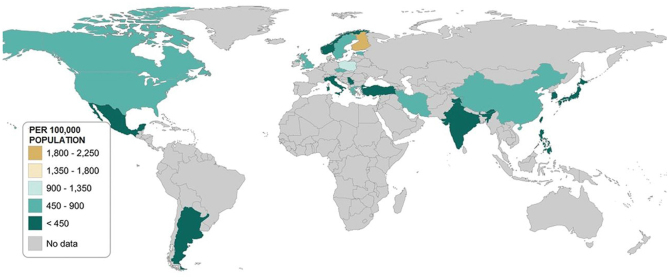
Adult period prevalence of rheumatoid arthritis (1955–2015) per 100 000 population, displayed globally. The map categorizes regions by prevalence rates: <450, 450–900, 900–1350, 1350–1800, and 1800–2250. The highest prevalence is observed in parts of North America and Northern Europe, whereas some regions in Asia and South America show lower prevalence rates.

**Figure 2.**
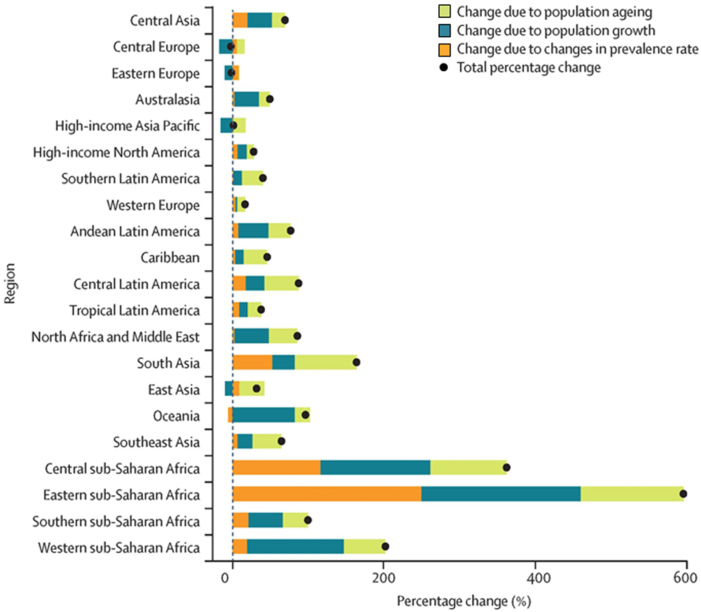
Projected percentage change in the number of prevalent rheumatoid arthritis (RA) cases by region from 2020 to 2050.

Rheumatoid arthritis (RA) is one of the most prevalent systemic autoimmune diseases characterized by inflammatory arthritis that can have extra-articular effects[1]. It is a chronic, symmetrical inflammation affecting synovial joints, caused by genetic (e.g., HLA-DR4, HLA-DR1) and environmental factors such as smoking. It typically affects smaller joints, progressively involving peripheral larger joints^[2,3]^. RA is a progressive disease with an increased rate of morbidity and mortality[4]. The annual incidence rate of RA is about 3 cases per 10 000 people globally, with a prevalence rate of roughly 1%, which rises with age (Fig. 1)^[5,6]^. The projected changes in the prevalence of RA cases from 2020 to 2050 vary significantly across different regions, as illustrated in Fig. 2[7]. The most substantial increases are anticipated in Central, Eastern, and Southern sub-Saharan Africa, driven primarily by population growth and aging, with additional contributions from changes in prevalence rates[7].

This disease typically begins between the ages of 35 and 60, characterized by periods of remission. However, it can also affect young children under 16, known as juvenile RA[8]. Prolonged joint inflammation can destroy bones and cartilage, weaken tendons and ligaments, and lead to painful deformities and erosions^[9,10]^.

Common symptoms of RA are morning stiffness in the affected joints lasting over 30 minutes, fatigue, fever, weight loss, tender and swollen joints that feel warm, and the presence of rheumatoid nodules under the skin[9]. RA with symptoms presentation of less than 6 months is referred to as early RA, whereas if the symptoms persist for longer than 6 months, it is referred to as established RA[9].

This narrative review aims to comprehensively synthesize current knowledge on RA, focusing on its multifactorial etiology, clinical manifestations, and the spectrum of disease progression. This review aims to identify key risk factors, genetic predispositions, and lifestyle elements contributing to the development and exacerbation of RA. The study will provide an integrated perspective on optimizing patient care by assessing the latest advancements in pharmacological treatments, including conventional DMARDs, biologics, and targeted gene therapies, alongside non-pharmacological interventions such as diet, physical activity, and complementary therapies. It also emphasizes the role of RNA-based therapies and new biomarkers, such as adenosine deaminases on the RNA (ADAR), which could guide more tailored treatment strategies. An essential contribution is the identification of the long-term safety concerns associated with these therapies, including immune suppression, metabolic disturbances, and treatment resistance, highlighting the need for careful monitoring. The review also underscores the practical implications for clinicians, advocating for personalized, precision-based approaches, proactive risk management, and consideration of cost-effectiveness and accessibility when selecting treatments. Ultimately, the objective is to bridge gaps in current understanding, highlight evidence-based practices, and propose future research priorities to enhance patient outcomes and quality of life.

### Etiology

The exact cause of RA is not fully known. It is theorized that in genetically predisposed individuals, an external trigger such as smoking, infection, or trauma initiates an autoimmune response. This response leads to synovial hypertrophy, chronic joint inflammation, and potentially extra-articular manifestations[5]. Smoking is the leading environmental risk factor for RA. Citrullination, the process where peptidyl arginine deiminases (PADs) convert arginine into citrulline, can be triggered by environmental factors such as smoking[11]. Genetic factors play a significant role in the risk, severity, and progression of RA.

### Juvenile idiopathic arthritis as a precursor to rheumatoid arthritis

Research highlights overlapping characteristics and trajectories between Juvenile Idiopathic Arthritis (JIA) and early-onset RA, with a potential continuum, especially in systemic or polyarticular JIA. Systemic-onset JIA, characterized by significant systemic inflammation, shares pathological features with RA, providing insight into a potential progression from JIA to RA in some instances[12]. Differentiating JIA from other inflammatory diseases remains essential, as JIA patients with rheumatoid factor (RF) positivity are often associated with a higher likelihood of developing an RA-like disease course in adulthood[13]. Although many JIA patients achieve remission within the first year of specialized treatment, some continue to experience persistent symptoms, which may indicate a more chronic trajectory similar to RA[14]. Studies examining JIA patients during the transition to adult care reveal sustained disease activity, reinforcing similarities with adult RA’s chronic nature and the need for extended management[15]. Furthermore, data from systemic JIA cohorts within the Childhood Arthritis and Rheumatology Research Alliance (CARRA) registry demonstrate variability in disease progression, suggesting that early intervention and tailored therapeutic approaches may be crucial in mitigating long-term disease progression[16].

The clinical diversity of JIA complicates diagnosis, particularly given its overlap with adult-onset RA. Diagnostic criteria require persistent arthritis for over 6 weeks in patients younger than 16, with subclassification based on the number of affected joints and extra-articular symptoms like fever, rash, and uveitis, which vary across subtypes[17]. Although a small subset of polyarticular JIA cases (2-5%) is RF-positive and closely resembles adult RA, other forms – such as systemic and enthesitis-related JIA – exhibit distinct clinical and laboratory characteristics[17]. RF and ANA tests, requiring positive results on two occasions 3 months apart, help distinguish seropositive polyarticular JIA from other subtypes. Radiographic imaging, while not essential for initial diagnosis, is used to monitor joint damage, as 15-20% of cases may eventually show radiographic changes[17].

Research on HLA-DRB1 alleles reveals critical genetic predispositions that may improve the diagnosis and understanding of JIA subtypes. Notably, the HLA-DRB108 allele significantly increases susceptibility to oligoarticular and polyarticular JIA forms, with an odds ratio (OR) of approximately 9.2 (95% CI: 6.7–13), indicating a strong genetic influence[18]. HLA-DRB101 and HLA-DRB104 are linked with RF-positive JIA, aligning RF-positive cases more closely with adult-onset RA. At the same time, HLA-DRB104 shows a protective effect in most other JIA subtypes (OR ~ 0.4 for oligoarticular JIA) but remains associated with systemic JIA (OR 1.9, 95% CI: 1.2–3.0)[18]. Additionally, HLA-DRB1*11 predisposes individuals to oligoarticular (OR 2.9, 95% CI: 1.9–4.3) and polyarticular JIA (OR 1.8, 95% CI: 1.2–2.5), underscoring the diagnostic utility of these HLA-DRB1 markers in distinguishing JIA subtypes[18].

Treatment for JIA has advanced significantly with the introduction of biologic agents, which provide targeted control over the disease’s inflammatory processes. Biologics inhibit specific cytokines, such as tumor necrosis factor (TNF)-α, interleukin (IL-1), and IL-6, which are central to JIA’s immune-driven pathology. Thus, they reduce the pro-inflammatory environment within affected joints, minimize synovitis, and prevent joint damage progression[19]. This targeted approach contrasts with traditional therapies that lack such specificity, offering a refined means of managing JIA’s complex immune mechanisms.

## Genetic markers of rheumatoid arthritis

RA is a multigenic disorder with 60% estimated heritability, of which 30% is contributed by HLA-DRB1 genes[20]. The primary genetic predisposition for this autoimmune disease is linked to major histocompatibility complex class II genes, with a significant focus on the prominent polymorphism in the HLA shared epitope (SE). Non-genetic factors contribute to the remaining risk[21]. The disease-linked alleles feature a conserved sequence of five amino acids known as the “shared epitope”[22]. According to the SE hypothesis, specific alleles with this conserved sequence contribute to the development of RA. This occurs through the improper presentation of autoantigens to T cells by antigen-presenting cells, leading to a T-cell-mediated autoimmune response^[22,23]^. These SE alleles have been strongly correlated with an increased susceptibility to RA, greater disease severity, and a heightened risk of developing extra-articular manifestations^[3,23,24]^.

### Non-HLA genetic factors in RA

Other than SE, the heritability of RA is strongly associated with loci like PTPN22, CTLA4 and PADI4^[25,26]^. Among the non-HLA-DRB1 loci, the PTPN22 gene is the most significantly associated with RA susceptibility. Together with the HLA-DRB1 alleles, these genetic factors are believed to account for approximately 50% of the genetic component of RA^[27,28]^. The PTPN22 protein is a lymphoid protein tyrosine phosphatase, an enzyme exclusive to immune cells. It plays a crucial role in modulating the responsiveness of T and B-cell receptors[29]. Despite the importance of this variant in the European population, it occurs rarely in Asian descent. Therefore, it is unlikely to contribute to RA pathogenesis amongst these populations^[30,31]^. Genetic markers for RA also include STAT4 and TRAF1/C5[32]. STAT4 is a gene involved in regulating and activating the immune system, with mutations also found in autoimmune conditions like lupus[20]. The TRAF1/C5 genes are significant contributors to chronic inflammation (Table 1)[32].

**Table 1:** Key genes associated with rheumatoid arthritis (RA) and their encoded proteins.

Gene	Encoded protein	Association with RA
HLA-DRB1	Class II human leukocyte antigen/major histocompatibility complex	Strongest association with RA, particularly HLA-DRB1*01, DRB1*04, and DRB1*10 alleles, which contain the shared epitope (SE).
PTPN22	Protein tyrosine phosphatase, non-receptor type 22	Associated with increased risk for RA, particularly in ACPA and RF positive RA.
TRAF1/C5	Tumor necrosis factor receptor-associated factor 1	Implicated in cell growth, proliferation, apoptosis, bone turnover, and cytokine activation.
STAT4	Signal transducer and activator of transcription 4	Involved in cytokine signaling, particularly IL-12 through JAK2, associated with both ACPA positive and negative RA.
PADI4	Peptidylarginine-deiminase 4	Mediates citrullination of proteins, strong association in Japanese and Korean cohorts, weaker in Caucasians.
IRF5	Interferon regulatory factor 5	-
FCGR	Fc gamma receptor	Key players in antigen presentation and inflammation, particularly FCGR3A (Fcγ receptor IIIA).
IL2RA, IL2RB	Interleukin 2 receptor alpha and beta	-
CD40	CD40	Member of the TNF-receptor superfamily, important in B-cell development and co-stimulation.
CCL21	CC chemokine ligand 21	-
CCR6	CC chemokine receptor 6	Expressed by Th17 cells, involved in IL-17 driven inflammation.

ACPA, anti-citrullinated protein antibodies; CCR6, CC chemokine receptor 6; CCL21, CC chemokine ligand 21; CD40, cluster of differentiation 40; DMARDs, disease-modifying antirheumatic drugs; FCGR, Fc gamma receptor; HCV, hepatitis C virus; HLA, human leukocyte antigen; IL-2RA/B, interleukin 2 receptor alpha/beta; IRF5, interferon regulatory factor 5; JAK, Janus kinase; PADI4, peptidylarginine-deiminase 4; PTPN22, protein tyrosine phosphatase, non-receptor type 22; RA, rheumatoid arthritis; RF, rheumatoid factor; SE, shared epitope; STAT4, signal transducer and activator of transcription 4; TRAF1/C5, tumor necrosis factor receptor-associated factor 1/C5.

### Contribution of genetic markers in personalized medicine

Understanding the genetic factors underlying RA could enable more aggressive and targeted treatments, ultimately improving patient outcomes. This approach reflects a broader trend in personalized medicine, where treatment is tailored based on an individual’s genetic makeup[33]. The current limitations in treating RA highlight the need for further research into the disease’s pathogenesis and the identification of new therapeutic targets. Fortunately, genetic molecules can serve as biomarkers, making identifying clinical subsets of RA easier. These biomarkers can help predict disease progression and guide treatment strategies[33].

## Biological markers for rheumatoid arthritis

### Inflammatory biomarkers

Inflammatory reactants like C-reactive protein (CRP) and cytokines, chemokines, and other reactants are increased when inflammation is activated. An increase in inflammation sets off a cascade of immunological reactions in RA. As a result, autoantibodies are overproduced, which causes RA patients’ levels of immunoglobulin M (Rheumatoid Factor) and anti-CCP (cyclic citrullinated peptide) to rise. Serum peptides that have undergone citrullination or other posttranslational modifications due to external stimuli present themselves as antigens to T cells and other immune cells, producing antibodies like anti-CCP. As a result, blood biomarkers for diagnosis include CRP, RF, and anti-CCP, which indicate RA’s inflammatory and immune response[34]. However, the current biomarkers for RA diagnosis have their limitations. For instance, RF has a sensitivity of 60–90% and a specificity of 85%. Anti-CCP, when combined with RF, can enhance the diagnostic accuracy of RA. In RA patients, anti-CCP has a greater ACCP positivity than RF positivity; however, there is no discernible difference between the two markers’ sensitivity and specificity. This underscores the need for new diagnostic biomarkers to complement the existing ones. The discovery of novel biomarkers holds the promise of revolutionizing the diagnosis and management of RA, particularly if they can identify seronegative (RF- and anti-CCP-negative) RA patients[34].

Beyond inflammation and immunity, new protein biomarkers that may be found by serum protein profiling are anticipated to reflect a variety of physiological alterations in RA. Recently, hematological parameters have emerged as potential indicators of systemic inflammation. These parameters, including the neutrophil-to-lymphocyte ratio, platelet-to-lymphocyte ratio, and lymphocyte-to-monocyte ratio, have shown promise in various medical fields, from cardiology to oncology. Their potential to serve as sensitive indicators of inflammation in autoimmune rheumatic diseases, including RA, is an intriguing area of research that warrants further exploration[35].

## Lifestyle intervention

### The role of smoking and alcohol

**Figure 3.**
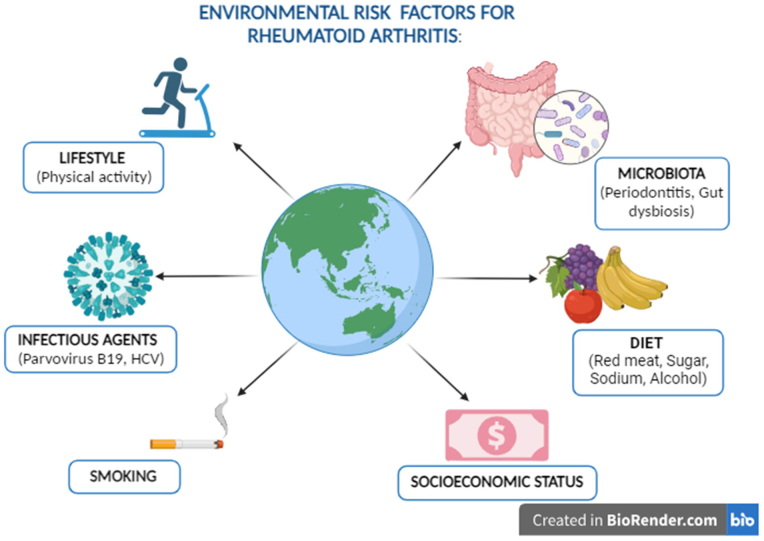
Environmental risk factors for rheumatoid arthritis include lifestyle, microbiota, diet, infectious agents, smoking, and socioeconomic status.

Environmental risk factors are significant determinants of population health and are crucial to managing RA (Fig. 3). Like other illnesses, RA is associated with smoking at its onset or worsening. Serendipity in a study with a different goal revealed the first indication that smokers have an elevated risk of RA[36]. It has since evolved into a well-described risk factor for RA. An extensive analysis of the dangerous compounds found in tobacco products indicates that smoking delivers a particular signal. It’s possible that smoking has a hereditary component that plays a specific function in initiating a particular subtype of RA[37]. A definite link between smoking and increased risk of RA in individuals with rheumatoid factor (RF) or anti-citrullinated protein antibodies (ACPA) has been established. A recent study in 2021 confirmed a positive correlation between smoking and a reduced risk of RA with higher alcohol consumption, demonstrating an inverse dose-response relationship. Among smokers, the protective effect of alcohol was particularly evident. Additionally, the combined use of alcohol and smoking was found to elevate the incidence of RA. Specifically, smoking was associated with a hazard ratio (HR) of 2.80 for the development of RA in non-drinkers, while among alcohol users, the corresponding HR was 1.45[38].

### Diet and nutrition

The exact etiology of RA is yet unknown; genetic predisposition accounts for a large portion of the risk, with the remaining portion presumably attributed to modifiable factors, including diet, exercise, and tobacco use[39]. A substantial amount of data indicates that dietary changes to enhance food quality are directly linked to improved health[40]. Food is a significant modifiable factor of chronic diseases. People with RA are interested in diet since it can help with their symptoms[41]. However, the research to date on the effectiveness of different dietary therapies for RA is still conflicting and unresolved. Dietary changes that support gut microbiota, reduce cytokine levels, and lower oxidative stress may influence RA outcomes[41].

Antioxidants in fruits and vegetables are known to help reduce oxidative stress levels and guard against the production of free radicals, which helps avoid inflammation[42]. Furthermore, studies indicate that oxidative stress may be linked to inflammatory CRP and TNF-α in RA patients[43]. Numerous studies have found that different dietary components have different anti-inflammatory qualities; food can affect the body’s inflammatory biomarkers[44]. The Mediterranean diet has several health advantages and is acknowledged as an anti-inflammatory diet[45]. Whole grains, seafood, fruits, vegetables, and extra virgin olive oil (EVOO) are essential elements of the Mediterranean diet.

By interfering with the expression of pro-inflammatory genes, immune cell activity, and the arachidonic acid cascade, these substances can have significant anti-inflammatory effects[46]. Furthermore, it has been demonstrated that fish containing omega-3 polyunsaturated fatty acids (PUFAs) affect the functions of lymphocytes and monocytes, which are essential for the immune system to eliminate invaders and regulate inflammatory pathways in chronic inflammatory diseases. Omega-3 PUFAs are widely recognized for their anti-inflammatory qualities and can lower inflammation by increasing macrophage autophagy[47].

Research on using polyunsaturated fats, especially omega-3 PUFAs, for treating RA dates to the mid-1980s. Several clinical trials have shown improvements in the pain and stiffness associated with RA in the mornings, as well as reductions in the amount of nonsteroidal anti-inflammatory drugs (NSAIDs) taken when omega-3 PUFA intake is increased[48]. Individuals who have RA also frequently report food intolerances and assert that certain meals, such as red meat, gluten-containing foods, and foods heavy in sugar and alcohol, exacerbate their symptoms[49]. It has been proposed that removing specific foods from the diet could help reduce the symptoms of RA. For instance, an animal-origin, high-fat Western diet has been positively correlated with the early start of RA and a burdensome disease state. This diet causes excess body weight and body fat. Potentially, pro-inflammatory mediators such as TNF-α, IL-6, and CRP are upregulated due to the interaction between chronic obesity and RA inflammation. This is a potential cause. Moreover, Western-style diets increase disease activity ratings and reduce the likelihood of reaching and maintaining illness remission[50].

Significant alterations in insulin sensitivity and blood lipid levels are linked to RA, particularly when the illness progresses, and metabolic abnormalities such as higher TC, LDL, and TG concentrations are present even in preclinical RA. While TC, HDL, LDL, and TG did not alter in either trial group, a negative correlation was seen between TG levels and the MedDietScore, which supports the MD’s cardio-protective benefits. Furthermore, patients receiving tailored MD intervention had significantly lower serum glucose levels than the controls, which suggests that MD has well-established beneficial effects on glucose regulation[50].

Additionally, prior research has also demonstrated a distinct negative association between dietary fiber intake and inflammatory markers, including plasma fibrinogen, TNF-α, high-sensitivity CRP (hs-CRP), and IL-6 levels, which are suggestive of RA[48]. Like whole grains, food items are deemed “whole” if they have equal proportions of germ, endosperm, and bran. Whole grains are known for having high concentrations of phytic acid, vitamin E, selenium, and antioxidants. These grains include wheat, corn, rye, whole rice, oats, barley, millets, sorghum, canary seed, and wild rice. It is thought that these ingredients help to lessen inflammation[48].

On the other hand, subtotal fasting is consuming limited amounts of carbs and energy, typically through vegetable juice and vitamin and mineral supplements. Fasting reduces the number and activity of CD4 + cells, which can restore the typical immune state linked to RA. RA is frequently associated with increased Th1 and Th17 lineage differentiation and CD4 + T cell activation. A brief period of fasting (7–10 days) can decrease T cell activation and have a temporary immunosuppressive effect. Fasting has been associated with evidence of reduced pain and inflammation (as measured by ESR and CRP); however, these advantages are only temporary and do not lead to long-term changes in disease activity[48].

### Physical activity and exercise

Compared to usual care, the “Plants for Joints” (PFJ) multidisciplinary lifestyle intervention has significantly improved disease activity in individuals with RA and pain, stiffness, and physical function in individuals with OA (osteoarthritis). The intervention involves a whole food plant-based diet, physical activity, sleep, and stress management. Under the direction of a research dietician, a multidisciplinary team with specialized knowledge in various aspects of lifestyle was responsible for developing and delivering the intervention in turn. Participants received one-on-one physical therapy consultations at the beginning and, if desired, at the end of the intervention. Coaches also provided further personalized guidance upon request[51]. Following each group session, participants were given a binder with homework activities, a Fitbit fitness tracker, recipes, a diet plan, and an optional fasting procedure.

Improved metabolic outcomes were seen in both the RA and OA groups, including notable weight, fat mass, HbA1c, and LDL cholesterol decreases. Owing to the PFJ intervention’s efficacy in enhancing disease-specific outcomes and (comorbidity risk factors), patients with RA, OA, and other non-communicable diseases may be able to employ it as an alternative therapy option[51].

Following bariatric surgery, a retrospective review of RA patients showed a significant decrease in inflammatory markers and a decrease in RA disease activity[52]. For 12 weeks, RA patients were placed on a hypocaloric diet consisting of 1000–1500 kcal per day in a randomized trial. In addition to losing 9.5 kg, these patients showed notable improvements in the disability index from the health assessment questionnaire and disease activity (DAS28)[52]. In addition to smoking habits, diet, obesity, marital status, and other individual characteristics, socioeconomic position is linked to many other factors that also affect the development of RA and the course of the illness[52].

### Chronic stress

Patients frequently believe that psychological stress is the root cause of their problem, and chronic psychosocial stress is mentioned as another potential component influencing the development of chronic diseases. According to psychoneuroendocrinological theories, long-term psychological stress can cause chronic illnesses by influencing the immune system through the corticotropic axis[52].

Psychosocial stress also appears to have an impact on the prognosis of established RA. In the randomized controlled CareRA-trial, individuals with early-stage arthritis who were placed in a group with a high psychosocial load had a higher chance of losing their state of remission[52].

### Socioeconomic status

A study reported at 6 months, increased DAS28 was linked to increasing deprivation when compared to the least deprived group[53]. In unadjusted models, a higher level of socioeconomic deprivation was similarly linked to a reduced chance of obtaining LDA (Low Disease Activity), remission, and EULAR (European Alliance of Associations for Rheumatology) response[53]. According to this study, a lower response to TNF inhibitors (TNFi) and a higher chance of treatment discontinuation were linked to socioeconomic hardship. A 1-year difference existed in the median time to discontinuance between the 20% most impoverished and 40% least deprived groups[53].

Socioeconomic status (SES) significantly impacts the clinical outcomes of RA, influencing disease severity, treatment efficacy, and patient adherence. Lower SES is associated with higher disease activity, as reflected by elevated DAS28 scores, and a reduced likelihood of achieving treatment milestones such as remission or LDA. Patients from disadvantaged backgrounds are less likely to respond effectively to biologic therapies like TNFi, potentially due to barriers such as limited access to healthcare, financial constraints, and difficulties in maintaining continuous treatment. Furthermore, lower SES is strongly linked to higher rates of treatment discontinuation, which can lead to disease flare-ups, increased disability, and long-term joint damage. These factors highlight the need for healthcare systems to address the disparities in RA management, ensuring that patients from all socioeconomic backgrounds have equal access to effective treatments and care.

## Conventional pharmacological treatments

### Symptomatic treatment with NSAIDS and glucocorticoids

**Figure 4.**
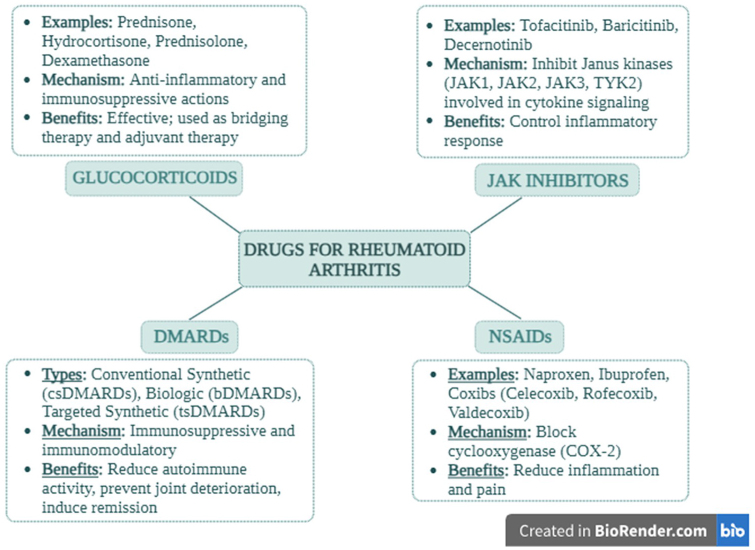
Overview of drugs used for rheumatoid arthritis treatment, categorized into glucocorticoids, JAK inhibitors, DMARDs, and NSAIDs.

The two approaches to managing RA that are now available, in compliance with ACR and EULAR guidelines, are disease-modifying antirheumatic therapy (DMARDs) and symptomatic treatment (NSAIDs and glucocorticoids [GCs]) (Fig. 4)[54]. NSAIDs and GCs are the mainstays of RA symptomatic treatment; however, after a thorough evaluation of the benefit-risk balance, mild opioid analgesics may also be taken into consideration for short-term pain management[55].

In the acute phase response, NSAIDs (naproxen, ibuprofen, and coxibs) decrease inflammation and pain. NSAIDs work pharmacologically by blocking the activity of cyclooxygenase (COX), particularly COX-2, which is elevated in inflammatory conditions. The suppression of prostaglandins can have significant side effects, including bleeding, gastrointestinal ulcers, renal failure, heart failure, rashes, disorientation, confusion, seizures, and others. Thus, it is essential to weigh the risk of injury. COX-2-selective NSAIDs (celecxib, rofecoxib, valdecoxib) can help prevent some of the adverse effects. In placebo-controlled trials with patients not receiving GC therapy, the efficacy of NSAIDs in treating RA has been shown[55]. Because of the complex mechanisms behind their anti-inflammatory and immunosuppressive actions, GCs (prednisone, hydrocortisone, prednisolone, and dexamethasone) are more potent and effective than NSAIDs. However, NSAIDs have a slightly better safety profile. Weight gain, water retention, muscle weakness, diabetes, bone weakening, and other long-term adverse effects are associated with GCs. They can therefore be given orally, intravenously, intramuscularly, or intra-articularly and have a brief half-life. GCs are used to treat RA in two main ways: first, as bridging therapy until the effects of DMARDs become apparent, and second, as an adjuvant therapy for active RA that does not go away even with DMARD use. The negative feedback in the regulation of the pulsatility of the hypothalamic-pituitary-adrenal axis makes it imperative that corticosteroid therapy not be suddenly stopped[55].

### Disease-modifying antirheumatic drugs (DMARDs)

Drugs called DMARDs are used to decrease autoimmune activity and postpone or stop joint deterioration to induce remission. Since DMARDs are slow-acting medications with a delayed onset of 6 weeks to 6 months, initiating the treatment as soon as possible is best to achieve better results. Three types of DMARDs have been identified: biologic DMARDs (bDMARDs), targeted synthetic DMARDs (tsDMARDs), and conventional synthetic DMARDs (csDMARDs). Patients with newly diagnosed RA are usually treated with csDMARDs as their first line of treatment. In cases when first-line therapy is not well tolerated or is ineffective, bDMARDs or tsDMARDs are advised. One benefit of tsDMARDs is that they can be taken orally, a feature that applies to the Janus kinase inhibitors (JAKi) class[55].

#### Conventional synthetic DMARDs (csDMARDs)

The class of medications known as csDMARDs is diverse and includes medications such as methotrexate (MTX), leflunomide (LEF), hydroxychloroquine (HCQ), and sulfasalazine (SSZ). These drugs are prescribed more frequently than agents with a lower efficacy and safety profile, like azathioprine, d-penicillamine, cyclosporine, minocycline, cyclophosphamide, and gold salts. Their modes of action result in an unfocused inhibition of the hyperactive immune system[56]. The 2021 ACR guideline for managing RA recommends that HCQ be conditionally indicated over other csDMARDs, SSZ be conditionally advised over MTX, and MTX be conditionally recommended over LEF for DMARD-naive patients with LDA[56].

#### Biologic DMARDs (bDMARDs)

When csDMARDs are unsuccessful or poorly tolerated, other treatment alternatives such as bDMARDs, tsDMARDs, biosimilars, or combination therapy are available. A more recent method for treating RA, bDMARDs offer targeted therapy on immune system components[56]. Based on their mode of action, bDMARDs are genetically modified protein molecules classified into multiple classes:

#### TNF-alpha inhibitors (TNFi)

TNF-alpha inhibitors, including Adalimumab, Certolizumab Pegol, Etanercept, Golimumab, and Infliximab (and their biosimilars), play a crucial role in RA treatment^[55,56]^. These agents inhibit TNF-alpha, a key cytokine in the inflammatory pathway of RA. By blocking TNF-alpha, these therapies disrupt the activation of several signaling pathways such as NF-kB, RANK, ERK, TPL2, and proapoptotic signaling. This inhibition reduces the stimulation of fibroblast-like synoviocytes (FLS) and osteoclastic activity, leading to decreased inflammation, prevention of joint damage, reduced arterial stiffness, and improved clinical outcomes. According to EULAR recommendations, TNFi is the first-line bDMARD therapy for patients who respond poorly to cDMARDs^[57,58]^.

#### IL-6 inhibitors

Monoclonal antibodies such as Tocilizumab and Sarilumab target IL-6 receptors (mIL-6R and sIL-6R). IL-6 is a pro-inflammatory cytokine involved in pannus formation and increased bone resorption. Inhibiting the IL-6 pathway helps to limit inflammation and slow disease progression[59].

Tocilizumab is a monoclonal antibody that inhibits IL-6. It is offered as an infusion on the pharmaceutical market and can be injected or subcutaneously applied. Regardless of the mode of administration, data from 14 phase 3 clinical trials revealed that tocilizumab has a low immunogenicity risk[60]. In RA patients who did not respond well to MTX therapy, tocilizumab therapy was found to be more beneficial than adalimumab monotherapy in terms of lowering signs and symptoms[34].

#### B-cell depleting agents

Rituximab, a genetically engineered chimeric monoclonal antibody, targets CD20-positive B lymphocytes. B cells play a crucial role in the autoimmune response by maintaining the inflammatory cascade through antigen presentation and proinflammatory cytokine production. Rituximab depletes B cell subpopulations via cytotoxicity, apoptosis, and growth arrest, reducing autoantibody production and the inflammatory response, resulting in clinical improvement.

Rituximab is a well-tolerated medication with no link to a higher risk of infection. An evaluation of the infection rates between rituximab and placebo was done using a fixed-effect meta-analysis. The trial results demonstrated that even at greater dosages, there is little chance of a significant infection when rituximab is used. Furthermore, prospective, noninterventional research showed that rituximab effectively treats RA. As such, patients who do not respond well to MTX or TNF-α inhibitors may consider rituximab as an option[61].

#### T-cell co-stimulation inhibitors

Abatacept, a fusion protein, inhibits T-cell co-stimulation by binding to CD80/CD86, interfering with its interaction with CD28. This mechanism reduces T-cell activation indirectly rather than blocking inflammatory proteins directly. Abatacept can be administered via intravenous infusion or injection. Other T-cell modulators like ALX-0061, Sirukumab, Clazakizumab, and Olokizumab are still undergoing trials[35]. According to the survey, abatacept and infliximab have comparable efficacy profiles, but abatacept has a better safety profile with fewer side events[35].

#### Other biologic therapies

Several other biologic agents are under investigation, including^[55,56]^:

- ***IL-1 Inhibitors***: Anakinra, Canakinumab, Rilonacept.
- ***B-cell Receptor Inhibitors***: Belimumab, Atacicept, Tabalumab.
- ***IL-12/23 Inhibitors***: Ustekinumab.
- ***IL-17 Inhibitors***: Ixekizumab, Secukinumab, Brodalumab.
- ***Granulocyte-Macrophage Colony-Stimulating Factor Inhibitors***: Mavrilimumab, Otilimab.
- ***RANKL Inhibitor***: Denosumab.

Scientific discoveries like bDMARDs have entirely changed how RA is treated. Numerous benefits have been documented in RA patients who did not respond well to csDMARDs. Therapeutic management aims to intervene swiftly and aggressively with the best medications. One of the main obstacles preventing patients from receiving bDMARDs is the high cost of biologics^[55,56]^.

### Emerging therapies and combination treatments

If a patient does not respond to tsDMARDs, bDMARDs, or csDMARDs, the monoclonal antibodies known as bDMARDs, which include adalimumab, infliximab, certolizumab, canakinumab, tocilizumab, sarilumab, and secukinumab, have specific targets that include TNF-α, IL-6, IL-1β, and IL-17. Additionally, tsDMARDs have targets. For instance, tofacitinib, baricitinib, filgotinib, upadacitinib, and decernotinib have JAK as their unique target[62]. Even though the DMARDs previously described have been highly effective in reducing RA, it remains an apparent reality that a considerable percentage of patients may encounter treatment failure, including limited efficacy and nonresponse. Rheumatologists advise RA patients to receive combination therapy for optimal therapeutic efficacy[62].

### Janus kinase inhibitors (JAKi)

The newest therapeutic approach to RA approved by the FDA and EMA involves the use of JAKi. Based on their level of selectivity, these compounds are categorized into two groups: the first group comprises low-selectivity inhibitors that block the signaling of a wide variety of cytokines.[63] In contrast, the second group includes inhibitors that may block specific signaling pathways. JAKs are cytoplasmic proteins that link membrane receptor-derived cytokine signaling to transcription factors called signal transducers and activators of transcription (STAT). This allows for the best possible control of the inflammatory response and can also be an essential therapeutic tool for autoimmune disorders[63].

Moreover, four members of the JAK family (JAK1, JAK2, JAK3 and tyrosine kinase 2, TYK2) and seven types of STATs (STAT1, STAT2, STAT3, STAT4, STAT5A, STAT5B, STAT6) can be targets for JAKi[64]. In addition to the excellent efficacy and safety profiles, other essential advantages of JAKi are their oral route of administration and lower production costs than those of bDMARDs.

The 2021 ACR guideline on RA treatment revised the advice on when to use JAKi instead of csDMARDs in cases of ineffectiveness. Additionally, patients who receive JAKi monotherapy may adhere to it more closely than those who receive multiple csDMARD therapies, even though the safety profile in this instance is lower[65]. Immunization is advised before starting treatment because immune system suppression can increase the risk of infections, particularly pulmonary ones[66]. A dose-dependent changes in lipid metabolism has been documented; however, this change has not been linked to an elevated risk of heart disease[55].

### Pharmacoeconomics

The availability of drug classes in public healthcare systems varies by nation and is determined by a thorough cost-benefit study. One helpful statistic for these pharmacoeconomic factors in a healthcare system is quality adjusted life years (QALYs)[64]. In the US, tofacitinib and baricitinib are around 68 and 32 times more expensive annually than MTX monotherapy, respectively, when the raw wholesale acquisition cost is used as a comparison[64]. A few economic analyses of tofacitinib were conducted in the United States, indicating either little additional expenses or even possible cost savings. As a second-line treatment, tofacitinib was found to be less expensive than other bDMARDs when administered as monotherapy to patients who were intolerant to MTX or in combination with MTX to patients who were intolerant to anti-TNF[64].

A study year’s total per year cost of reimbursed medications for RA therapy came to €81 206 363.70[67]. Nearly 70% of the entire expenditure (€52 732 142.18–64.94%) went toward biologics. In particular, over half of the total expenditure (48.92%, €39 724 489.71) was spent on anti-TNF therapy, with or without corticosteroids/MTX, and 16.02% (€13 007 652.47) was spent on other biologics, with or without corticosteroids/MTX[64]. The mean yearly cost per patient for anti-TNF monotherapy treatment was €7681. When methotrexate and anti-TNFs were added, this increased to €8488.19[64].

### Limitations

While bDMARDs and JAKi have significantly advanced the treatment of RA, there are several notable limitations associated with these therapies. One of the primary challenges is the high cost. Biologic therapies, such as TNF inhibitors (TNFi), IL-6 inhibitors, and JAKi, are significantly more expensive than csDMARDs. The cost of biologic treatments can be a significant barrier to access, particularly in public healthcare systems or for patients without comprehensive insurance coverage. Even though JAKi tend to be less expensive than biologics, they still represent a substantial cost burden when compared to older, more affordable therapies like MTX. This economic disparity can limit widespread use and delay timely treatment, which is crucial for managing RA effectively.

Both biologics and JAKi suppress the immune system, which increases the risk of infections, particularly respiratory and opportunistic infections. These therapies can leave patients more vulnerable to severe infections, which may complicate disease management, especially in older patients or those with comorbidities. While JAKi offer a more convenient oral administration route than biologics, they are not without their own risks. Notably, JAKi have been associated with dose-dependent changes in lipid metabolism, though studies have not conclusively linked these changes to a higher risk of cardiovascular disease. The long-term safety profiles of JAKi, particularly regarding immune function, remain a subject of ongoing research. Despite their efficacy, biologics and JAKi do not work for all RA patients. A significant proportion of patients may experience inadequate responses or no response to these therapies, necessitating either alternative or combination therapies. This inconsistency highlights the complexity of RA treatment and underscores the need for personalized medicine to identify the most appropriate therapy for each patient. Additionally, biologics, such as TNFi and IL-6 inhibitors, can sometimes trigger immune reactions, including the formation of antibodies against the drug itself. This immunogenicity can reduce the effectiveness of treatment over time, potentially leading to therapy failure and requiring a switch to another biologic or JAKi.

JAKi are administered orally, whereas many biologics require injections or infusions, which can be inconvenient and may result in lower patient adherence. Regular subcutaneous or intravenous administration of biologics can be burdensome for patients, especially those requiring frequent dosing. This inconvenience may affect long-term adherence to treatment regimens, potentially reducing the overall effectiveness of the therapy. Lastly, while biologics and JAKi are generally well tolerated, they are associated with a range of side effects that can impact patient quality of life. For instance, TNFi have been linked to an increased risk of certain cancers, while JAKi can cause liver enzyme elevations and affect blood cell counts. Though these side effects are relatively rare, they can be severe and may require discontinuation or modification of treatment. The long-term safety of these therapies, especially concerning their impact on organ systems such as the heart and lungs, is still under investigation. Continued research into more affordable, effective, and safer treatments is essential to improve outcomes for RA patients, particularly those who do not respond adequately to current therapies.

## Non-pharmacological therapies

Despite advancements in pharmaceutical treatments, many RA patients still struggle to meet treatment goals. RA, characterized by chronic inflammation and complexity, necessitates a holistic management strategy. Non-pharmacological therapies (NPTs) appear to have a multifaceted, all-encompassing impact on the immunological, neurological, and endocrine systems[57]. Combined with specific pharmacotherapies, they can significantly improve the course of treatment for RA. Physical activity lowers the risk of inflammatory rheumatic diseases and is vital for managing RA comorbidities, decreasing the risk of cardiovascular diseases, and also enhancing sleep quality and duration[68].

Many forms of physical therapy and hydrotherapy have been researched as non-pharmacological treatment alternatives. Numerous groups of RA patients have undergone cryotherapy studies; both local and whole-body cryo-stimulation showed favorable effects on clinical and laboratory measures, and when paired with physical activity, cryotherapy can effectively reduce pain and disease activity[69]. Laser acupuncture, neuromuscular electrical transmission, and transelectrical nerve stimulation (TENS) have demonstrated some promising benefits^[70-72]^. A national survey of US rheumatologists found that 54% thought acupuncture helped manage pain[73].

Cognitive behavioral therapy (CBT) helps alter negative thought patterns to improve symptoms and disability in RA patients. It has been shown to offer small-to-moderate benefits in reducing impairment and fatigue and enhancing self-efficacy. Positive emotions also encourage good social interactions, which are inversely linked to physical health. They alleviate chronic pain and reduce its impact on RA patients’ functioning and well-being. Lower levels of proinflammatory cytokines in the blood are associated with positive emotion^[74,75]^.

Certain plants and their components have been shown to have therapeutic and preventative effects on the treatment of RA. Chinese herbal remedies such as Radix Gentianae Macrophyllae, Radix Stephaniae Tetrandrae, Caulis Sinomenii, and Caulis Lonicerae are being explored for new RA treatments due to their significant historical use[76]. Research highlights their active components – gentiopicroside, tetrandrine, sinomenine, and chlorogenic acid – as potentially beneficial against RA.

Certain SIRT1 gene variations (rs3740051, rs7069102, and rs1467568) have been linked to RA susceptibility in the Chinese Han population, according to a recent study[76]. Natural medications (like resveratrol, baicalin, and α-mangiferin) in the RA model inhibit the transcriptional activity of NF-κB by up-regulating SIRT1 expression. This suppresses the expression of TNF, IL-6, and IL-1β, as well as the proliferation, invasion, and migration of FLSs, all of which benefit RA[77]. According to a recent meta-analysis, coffee drinking, in general, showed a positive correlation with the risk of RA. The scientists hypothesized that additional chemicals used in coffee processing and production, besides caffeine, may also reduce the incidence of RA[78].

RA patients’ quality of life, perception of their condition, and psychological elements (such as sadness, anxiety, and stress) can all be improved by mindfulness training, which also helps to lower disease activity, physical disability, and inflammation[79].

## Emerging strategies in the treatment of RA

In recent years, personalized treatment strategies for RA have increasingly focused on tailoring therapies to individual patient profiles. Advances in genomics, biomarkers, and biologic therapies have enabled a shift from one-size-fits-all approaches, allowing for more precise treatment based on specific disease characteristics and patient responses. The success of mRNA vaccines, which have demonstrated effectiveness in rapidly inducing immune responses against infectious agents such as SARS-CoV-2, offers promising potential for RA management[80]. Future RA therapies could involve personalized mRNA-based approaches to target the immune cells and cytokines responsible for joint inflammation and damage[80]. Such strategies could allow for more accurate modulation of autoimmune responses, reducing the side effects typically associated with traditional biologics and enhancing the specificity and efficacy of treatments for RA patients.

The development of nano-vaccines, including needle-free and combinatory formulations, further holds promise for advancing RA treatment[81]. These innovative vaccine delivery systems can enhance the stability and bioavailability of antigens, facilitating more targeted immune responses with fewer adverse effects compared to conventional biologic therapies[81]. By directly delivering RA-specific antigens to the immune system, nano-vaccines could reduce inflammation and joint damage[81]. As research progresses, personalized nano-vaccine-based approaches may offer more effective, less invasive alternatives to existing treatments, further advancing the field of RA therapeutics.

Zoonotic diseases, which are transmitted from animals to humans, present significant public health challenges, significantly as climate change accelerates their transmission. This growing concern also affects chronic conditions such as RA[82]. The increasing frequency and geographic spread of zoonotic pathogens due to environmental changes could have direct implications for RA patients, whose immune systems are already compromised[82]. As climate change alters ecosystems and brings humans closer to wildlife, new forms of cross-species transmission may trigger autoimmune responses or exacerbate pre-existing conditions like RA. The One Health approach recognizes the interconnectedness of human, animal, and environmental health and offers a comprehensive framework for managing RA in climate change[82]. This approach addresses zoonotic disease transmission and provides insights into how environmental factors may influence immune system dysregulation, potentially informing RA pathogenesis and treatment strategies.

Stem cell therapy is emerging as a promising treatment for RA, potentially regenerating damaged tissues and modulating immune system function[83]. By utilizing the regenerative properties of stem cells, mainly induced pluripotent stem cells (iPSCs), researchers are exploring novel ways to repair joint damage, reduce inflammation, and potentially reverse the autoimmune processes underlying RA[83]. This approach could provide a new avenue for more effective and long-lasting treatment options for RA patients.

## Patient-centered care and decision-making

A patient-centered approach is a vital step in treating RA, as it includes tailoring treatment plans to each patient’s preferences, values and lifestyle factors[84]. Studies show that RA patients don’t concern themselves about taking multiple pills for their treatment but rather consider the adverse effects of RA minor as compared to treatment benefits[84]. New research results show that socioeconomic factors affect patients more than the comorbid conditions[85]. The outline of the research was “The impact of socio-economic status in RA.” this research showed that low socioeconomic status (SES) was directly connected to lower-quality clinical outcomes[86]. Appropriate RA treatment should be selected after shared decision-making between healthcare providers and patients to align with patients’ personalized goals^[84,87]^. Patients should be involved in each step while identifying the proper treatment. Following a patient-centered strategy, shared decision-making, and engaging tools, RA patients can be empowered to be more involved in their treatment and achieve maximum optimal health benefits[85].

## Technological advancements and digital health

With the new era of advancement of IT, technology is making its way into healthcare departments by incorporating technology for diagnosing and treating diseases. Integrating telemedicine, remote monitoring, and electronic health records (EHR) is enhancing access to care and monitoring disease progression in RA[88]. Telemedicine is the remote diagnosis and treatment of patients using telecommunication technology. Telemedicine is increasing in rheumatology, which allows patients to be consulted virtually by professionals[89]. This would benefit patients living in remote areas or with limited mobility[89]. High patient satisfaction has been reported in some studies regarding telemedicine[89]. Remote monitoring of Rheumatoid Arthritis (REMORA) analysis showed that ePROMs (electronic patient-reported outcome measures) tools better view RA by precisely recording the varying symptoms[89]. EHRs are now the standard practice for keeping records of patients as they allow access to patient records to all physicians and healthcare providers, which enhances patient care and can also prevent duplicate testing[89]. A recent randomized clinical trial showed that using an intelligent disease management (SSDM) app for patient reports enhanced disease control[90]. SSDM app allowed patients to report their symptoms better and allowed patient-physician interaction[90].

Mobile health (mHealth) apps and wearable devices have evolved to detect conditions like RA. A trial known as the GSK wearable-PRO study was a 14-day observational study that showed that sensor-based measures recorded by iPhone and Apple Watch have proved high accuracy in differentiating between a fit person and an RA patient[89]. The use of wearable devices and smartphone apps outside of a clinical environment has made following RA disease activity easier.

Artificial intelligence (AI) has shown phenomenal results across different fields of life and has now been used to improve healthcare. AI helps make personalized treatment plans for RA patients. AI helps understand RMDS, patient and risk stratification, and outcome prediction. AI also helps discover new drug targets and treatment options for RMDs[88]. A study conducted by a Japanese research team used machine learning algorithms to create a model that predicted the transformation of undifferentiated arthritis into RA, resulting in a perfect early diagnosis[91]. Numerous studies have been conducted using AI to diagnose RA. AI has also optimized personalized treatment plans for RA. A survey of AI in RA showed that machine learning (ML) models may be used to identify clinical features essential in determining patient treatment plans[92]. For a study that used ML, predicted flares in RA patients using physical activity data, ultrasound and blood test reports[92].

While technological advancements like telemedicine, remote monitoring, AI, and wearable devices hold great potential for improving RA management, several challenges remain. First, the need for high-quality, heterogeneous datasets for AI models poses difficulties in obtaining accurate and reliable data, especially from diverse patient populations. Regulatory hurdles, including FDA approval and compliance with safety standards, slow the integration of AI tools into clinical practice. Additionally, disparities in access to technology, especially among underserved or older populations, may exacerbate existing healthcare inequalities. Data privacy and security concerns also arise with the digital storage of sensitive health information. Furthermore, the clinical validation of AI tools and their seamless integration into existing workflows require rigorous testing and clinician training. Patient engagement and adherence to digital health tools are crucial but often challenging, as is the financial burden of these technologies on patients and healthcare systems. Addressing these limitations will be essential to maximizing the benefits of digital health in RA care^[91,92]^.

## Recent researches and future directions

RA is a chronic inflammatory disease. Despite ongoing treatment, including DMARDs, NSAIDs, and GCs, patients still experience physical impairment, rendering the disease incurable[93]. Despite the success of biological agents to treat RA, such as the use of anti-inflammatory molecules, inhibition of pro-inflammatory signaling pathways and carrying out immunomodulatory therapies utilizing anti-inflammatory cytokines, the use of all these is often limited due to the rise of economic burden, gastrointestinal and respiratory complications and some other cutaneous reactions[94].

As conventional methods for the treatment of RA have a limited success rate, Gene therapy is being explored as the potential treatment for the future. Gene therapy for RA is being considered as a specific approach used in therapy for patients who are suffering from remission of RA and cannot respond to conventional treatment methods such as DMARDs and other biological agents[95].

Recent studies on RA unveiled an intricate network of RNA metabolic pathways that contribute to treating the disease. It has been evident that integration of modified ribonucleotides into small interfering RNA (siRNA) molecules shows an antigenic immune response while preserving RNA interference (RNAi) effectiveness[93]. Moreover, the increased occurrence of ADARs has been shown in the synovial membrane of RA patients, which leads to an increase in adenosine-to-iodine editing of messenger RNA (mRNA). This biochemical modification on amino acids of the protein has been shown in gene expression; including the extracellular matrix-degrading enzyme cathepsin S. Increased RNA modification of A-to-I editing of cathepsin S mRNA has been associated with protein expression in synovial tissue of RA patients. Hence, it plays a vital role in RA progression[96]. Fascinatingly, ADAR is used as a potential biomarker for predicting TNFi response, as it has been observed that effective treatment with TNF is reduces the expression of ADAR and its activity[96]. Furthermore, TNF-alpha (pro-inflammatory cytokine) induction of ADAR expression and activity in RA indicates significant implications for therapy, including reducing inflammation, modulating immune response and more[96].

RA is a complex autoimmune disease, and despite its pathobiology being challenging to understand, various exogenous and lifestyle factors are responsible for its pathogenesis. These include tobacco smoking, unbalanced diet, obesity, unhealthy lifestyle, persistent stress etc[96]. This sedentary lifestyle, including other factors such as metabolic syndrome, may cause some other long-term diseases resulting in chronic inflammation. Hence, people with diabetes mellitus and cardiovascular diseases have more severe symptoms of RA than in the general population[97]. A multimodal intervention named “Plants for joints” has been designed recently to integrate a whole food-plant-based diet, physical activity, mindfulness-based stress reduction activities, and sleep hygiene habits. As this program uses non-pharmacological therapies for RA management, patients can gain quality of life, developing no side effects[96]. Ongoing research says that a healthier lifestyle is associated with a lower risk of RA. This is observed by a Nurses’ health study, which diagnosed a total of 1219 incident RA cases, and women with healthy lifestyle factors had the lowest risk^[98]^.

Personalized treatments for RA, such as biologics, JAKi, and gene therapies, offer significant advancements by targeting specific immune pathways to provide more effective and tailored disease management. However, these therapies come with notable long-term risks. Biologics and JAKi, while highly effective, can suppress the immune system, increasing the risk of infections and potentially leading to complications like gastrointestinal issues, respiratory diseases, and metabolic changes such as elevated cholesterol levels. Gene therapies, though promising, raise concerns about the long-term safety of genetic modifications, with the potential for unintended alterations to immune or cellular processes. Moreover, not all patients respond to these treatments; some may develop resistance over time, limiting their effectiveness. The high cost of personalized therapies remains a significant barrier to accessibility, particularly in low-income settings, and poses a financial burden on healthcare systems. Balancing the potential benefits of these advanced treatments with their associated risks will ensure their long-term success and accessibility for RA patients worldwide.

## Conclusion

RA represents a complex interplay of genetic, environmental, and lifestyle factors, which drive the disease’s onset, progression, and variability in patient outcomes. Advancements in understanding genetic markers, such as HLA-DRB1 alleles and other non-HLA loci, have provided valuable insights into the genetic basis of RA, laying the groundwork for more precise, targeted therapies. These markers offer predictive value in identifying individuals at higher risk and serve as essential tools for tailoring treatment plans, marking a significant shift toward personalized medicine.

Incorporating lifestyle interventions, particularly diet and physical activity – into RA treatment regimens demonstrates the importance of a holistic approach. Diets rich in anti-inflammatory compounds, such as the Mediterranean diet and omega-3 fatty acids, have shown potential in mitigating inflammation. At the same time, physical activity contributes to overall health improvement and reduces RA-associated comorbidities. Together, these non-pharmacological measures offer significant, sustainable benefits when used alongside traditional treatments, addressing symptom management and disease progression.

Emerging pharmacological therapies, including JAKi and biologic DMARDs, represent groundbreaking steps forward by targeting specific inflammatory pathways with greater precision, providing options for patients unresponsive to conventional treatments. Despite these advances, challenges remain, including the high cost of biologics, treatment-related side effects, and issues with patient adherence. Meanwhile, technological advancements, particularly AI, are revolutionizing RA management by enabling early diagnosis, risk stratification, and personalized treatment planning. These technologies promise to transform clinical practice, allowing physicians to manage the disease based on individualized patient profiles proactively.

The future of RA management lies in a more integrated and personalized approach, combining genetic insights, advanced pharmacological treatments, and lifestyle management. This holistic model can improve clinical outcomes, reduce disease progression, and enhance long-term patient quality of life. However, several key areas require further research: validating the clinical application of genetic markers for early diagnosis and personalized treatment, refining lifestyle interventions and assessing their long-term impact on disease outcomes and addressing the barriers to widespread adoption of AI and digital health technologies in clinical practice. Researchers and clinicians must work collaboratively to explore these areas, ensuring that emerging therapies and technologies are both practical and accessible and address the diverse needs of all RA patients. By continuing to push the boundaries of research and clinical innovation, we can improve the quality and outcomes of RA care for patients worldwide.

## Data Availability

Not available.
